# Mechanically Referenced Early Acoustic Emission Indicators of Thermally Modified Deformation in S235 Structural Steel

**DOI:** 10.3390/ma19183958

**Published:** 2026-09-17

**Authors:** Anna Adamczak-Bugno

**Affiliations:** Faculty of Civil Engineering and Architecture, Kielce University of Technology, Av. 1000-An. of Polish State 7, 25-314 Kielce, Poland; aadamczak@tu.kielce.pl

**Keywords:** acoustic emission, S235 structural steel, high-temperature exposure, post-fire assessment, cumulative AE counts, residual mechanical properties, pre-yield behaviour, stage-resolved analysis

## Abstract

**Highlights:**

- Stage-resolved acoustic emission (AE) reveals thermal-history effects before macroscopic yielding.
- A 14% decrease in yield strength accompanied a 210% increase in pre-yield AE counts.
- Similar final cumulative counts may correspond to distinctly different AE histories.
- Thermal exposure redistributes AE activity between successive deformation stages.
- Pre-yield AE descriptors complement conventional residual strength parameters.

**Abstract:**

Post-fire assessment of structural steel typically relies on residual mechanical properties, although thermally induced changes in deformation behaviour may emerge before conventional strength parameters are clearly affected. This study examines whether mechanically referenced acoustic emission (AE) descriptors can reveal such changes in S235 steel after exposure to 700, 900, and 1100 °C for 40, 80, and 120 min, followed by air cooling. Thirty specimens were subjected to tensile testing with two-channel AE monitoring. The two channels were recorded independently and were not combined. Channel 2 was used consistently for quantitative comparisons, whereas channel 1 served as a control of signal transmission and sensor coupling. Median cumulative AE counts were evaluated at 75% of the lower yield load, the lower yield point, ultimate tensile strength, and fracture. Thermal exposure altered both the magnitude and distribution of AE activity, with the greatest differences occurring before or near yielding. After exposure to 900 °C for 120 min, the mean lower yield strength decreased by approximately 14%, while pre-yield AE counts increased by approximately 210% relative to the reference condition. Specimens exposed to 1100 °C for 40 min showed similar final AE counts to the reference specimens but markedly different distributions across the loading stages. Stage-resolved AE analysis therefore provides information complementary to residual mechanical properties and final AE counts. Although the proposed pre-yield descriptor is retrospective and cannot be applied directly as a field-diagnostic threshold, it provides a sensitive experimental basis for comparing thermally modified deformation histories and for future development of independently defined AE-based assessment criteria.

## 1. Introduction

Structural steel is one of the principal load-bearing materials used in contemporary buildings and infrastructure because of its high strength, ductility, and versatility in structural design. During fire exposure, however, its mechanical response changes considerably as temperature increases. Reductions in elastic modulus, yield strength, and tensile strength may lead to excessive deformation, loss of stability, and ultimately structural failure [1,2,3]. Although part of this reduction may be recovered after cooling, sufficiently severe thermal exposure can produce persistent changes in the microstructure and residual mechanical properties of structural steel [4,5,6,7].

Post-fire assessment therefore represents a different problem from the evaluation of steel behaviour during fire. The residual state of the material is determined not only by the maximum temperature reached but also by the exposure duration and cooling conditions. Depending on the steel composition and thermal history, high-temperature exposure followed by cooling may induce recovery, recrystallization, grain growth, redistribution of residual stresses, and, under sufficiently severe conditions, phase transformations affecting the subsequent deformation and fracture behaviour. Consequently, steels exposed to similar maximum temperatures may exhibit different residual mechanical responses after cooling [5,6,8,9]. Recent studies have further emphasized the importance of history-dependent descriptions of metallic-material behaviour and the use of constitutive modelling to account for the effects of preceding thermal and mechanical conditions on subsequent deformation [10]. Such developments reinforce the need for complementary experimental descriptors capable of identifying changes in material behaviour that may not be fully represented by individual residual strength parameters.

Conventional post-fire assessment is predominantly based on visual inspection, geometrical measurements, mechanical testing, hardness measurements, and microstructural characterization. Among laboratory methods, uniaxial tensile testing provides direct information on residual yield strength, ultimate tensile strength, elastic response, and ductility and therefore remains one of the fundamental approaches for quantifying thermally induced material degradation [2,5,7,9]. These parameters are indispensable for assessing residual load-bearing capacity. Nevertheless, they primarily describe the macroscopic outcome of degradation. Changes in the mechanisms governing deformation may already be present before they become clearly reflected in global stress–strain characteristics or conventional strength parameters.

Acoustic emission (AE) monitoring offers a complementary perspective because it records transient elastic waves generated by active processes occurring within a material during loading. In metallic materials, AE activity may accompany dislocation motion, local plastic deformation, microcrack initiation and propagation, and other irreversible microstructural processes [11,12]. Unlike conventional mechanical measurements, which describe the global response of a specimen, AE is sensitive to localized processes developing during deformation. This characteristic makes AE particularly attractive for detecting alterations in material behaviour at stages at which corresponding changes in the macroscopic mechanical response may still be limited.

The diagnostic potential of AE has been extensively investigated in concrete, reinforced concrete, composites, and metallic materials [13,14,15]. In steels, AE has been applied to the investigation of discontinuous yielding, tensile deformation, fatigue damage, and successive stages of plastic deformation and fracture [16,17,18,19]. AE responses of stainless steel during elevated-temperature tensile loading have previously been investigated [20]. Related studies have demonstrated the applicability of AE descriptors to mechanically tested prestressing steel and cement-based materials [21,22,23]. However, considerably less attention has been given to the AE behaviour of previously heated and cooled structural steel during subsequent tensile loading at ambient temperature. In particular, the acoustic response generated during reloading of previously heated and cooled structural steel has received considerably less attention than conventional residual mechanical properties. As a result, it remains insufficiently understood whether changes in AE activity can serve as early indicators of thermally induced mechanical degradation.

This distinction is important from a diagnostic perspective because thermal exposure modifies the material state from which subsequent mechanical loading begins. Consequently, specimens exhibiting relatively similar portions of their macroscopic tensile response may differ in the onset, intensity, and distribution of AE activity generated during deformation. Acoustic emission may therefore provide information not only about advanced damage or impending fracture but also about changes in deformation processes developing before macroscopic yielding becomes apparent.

Among the large number of AE descriptors available, cumulative counts provide a straightforward means of tracking the development of acoustic activity during loading. However, their interpretation depends strongly on the mechanical stage at which they are evaluated. A final cumulative value recorded at fracture integrates activity generated throughout the loading history and may therefore conceal differences in its temporal distribution. Mechanically referencing cumulative counts to characteristic stages of the tensile response may preserve information on whether AE activity is preferentially generated before yielding, during plastic deformation, or during localization and final fracture [15,23].

Cumulative AE counts were selected as the primary descriptor because the objective of the study was to determine when detectable acoustic activity accumulated relative to mechanically defined stages of tensile deformation, rather than to classify individual AE source mechanisms. Counts provide a direct measure of successive threshold crossings and enable continuous tracking of the development of recorded acoustic activity throughout loading. When the acquisition threshold, hit-definition parameters, sensor arrangement, coupling procedure, specimen geometry, and loading conditions are maintained consistently, cumulative counts can be used as a comparative descriptor of AE evolution within a controlled experimental programme. They are not, however, treated here as an intrinsic material property or as a direct measure of released energy.

In particular, limited attention has been given to whether changes in AE activity can be identified at mechanically defined pre-yield stages when corresponding differences in conventional residual mechanical parameters remain comparatively limited. It also remains unclear whether thermal exposure primarily changes the total amount of acoustic activity or redistributes this activity between successive stages of deformation.

The novelty of the present study lies in the mechanically referenced, stage-resolved evaluation of AE activity in thermally exposed S235 structural steel. Instead of relying primarily on the total acoustic activity accumulated up to fracture, cumulative AE counts are evaluated at characteristic stages of the tensile response, including a normalized pre-yield level, the lower yield point, ultimate tensile strength, and fracture. Relative changes in pre-yield AE activity are compared directly with changes in residual lower yield strength, while normalized cumulative-count ratios are used to quantify the redistribution of AE activity over the loading history. This framework makes it possible to distinguish changes in the timing of AE activity from changes in its final cumulative magnitude.

Accordingly, the present study investigates whether mechanically referenced AE activity reveals thermally induced modifications in the deformation behaviour of S235 steel during the pre-yield stage and whether these changes provide information complementary to conventional residual mechanical properties. Specimens were subjected to controlled exposure at 700, 900, and 1100 °C for different durations, cooled in air, and subsequently tested in uniaxial tension with simultaneous AE monitoring.

The study addresses two principal research questions: (i) whether thermally induced differences in AE activity are detectable at a mechanically normalized pre-yield stage when changes in residual mechanical properties remain comparatively limited, and (ii) whether thermal exposure changes the distribution of cumulative AE activity between the pre-yield, yielding, UTS, and post-UTS stages. The mechanically referenced pre-yield parameter used in this study is retrospective because the corresponding load level is determined from the yield load of each specimen. It is therefore evaluated as a stage-resolved comparative descriptor rather than as a prospective field-diagnostic threshold.

Accordingly, the present study does not propose *N*_cum,75YS_ as an on-site diagnostic threshold. Its purpose is to test, under controlled laboratory conditions, whether thermally modified material states produce distinguishable AE histories before macroscopic yielding. Translation of this concept to structural assessment would require a prospective AE descriptor referenced to an independently known stress, strain, or service-load level.

## 2. Materials and Methods

### 2.1. Material, Reference Microstructure, and Specimen Preparation

The experimental investigation was conducted on S235 low-carbon structural steel in the as-received condition and after controlled high-temperature exposure. The reference material represented hot-rolled steel in the delivery condition without additional heat treatment. Flat tensile specimens were cut from hot-rolled steel plate and prepared in accordance with the requirements for tensile testing of metallic materials.

The material was identified as S235 structural steel on the basis of the available material documentation. S235 is a low-carbon structural steel typically characterized by a ferritic–pearlitic microstructure and a relatively low carbon content. For reference, the chemical composition of S235 steel is generally characterized by a carbon content not exceeding approximately 0.17–0.20 wt.%, together with limited additions of Mn, Si, P, and S, depending on the product form and applicable grade specification [24]. These values are provided only as a general compositional reference for the S235 steel grade and do not represent a chemical analysis of the particular steel batch investigated in the present study. Detailed chemical-composition data were not available; consequently, the results are interpreted as specific to the investigated steel product and should not be generalized to all heats and products classified as S235.

The specimens had a symmetrically tapered reduced section. The width decreased gradually from 10 mm near the transition radii to a minimum of 6 mm at the centre of symmetry of the gauge section. All specimens had the same nominal geometry, an initial thickness of approximately 5 mm, and an extensometer gauge length of 25 mm. The cross-sectional area used to calculate engineering stress was determined at the minimum-width section located at the centre of symmetry of the specimen.

All tensile specimens were cut in the same orientation relative to the rolling direction, parallel to the rolling direction.

The dimensions of each specimen were measured using a precision caliper and micrometer with a resolution of 0.01 mm. The individually measured dimensions were used to determine the initial cross-sectional area for the calculation of engineering stress.

**Figure 1:**
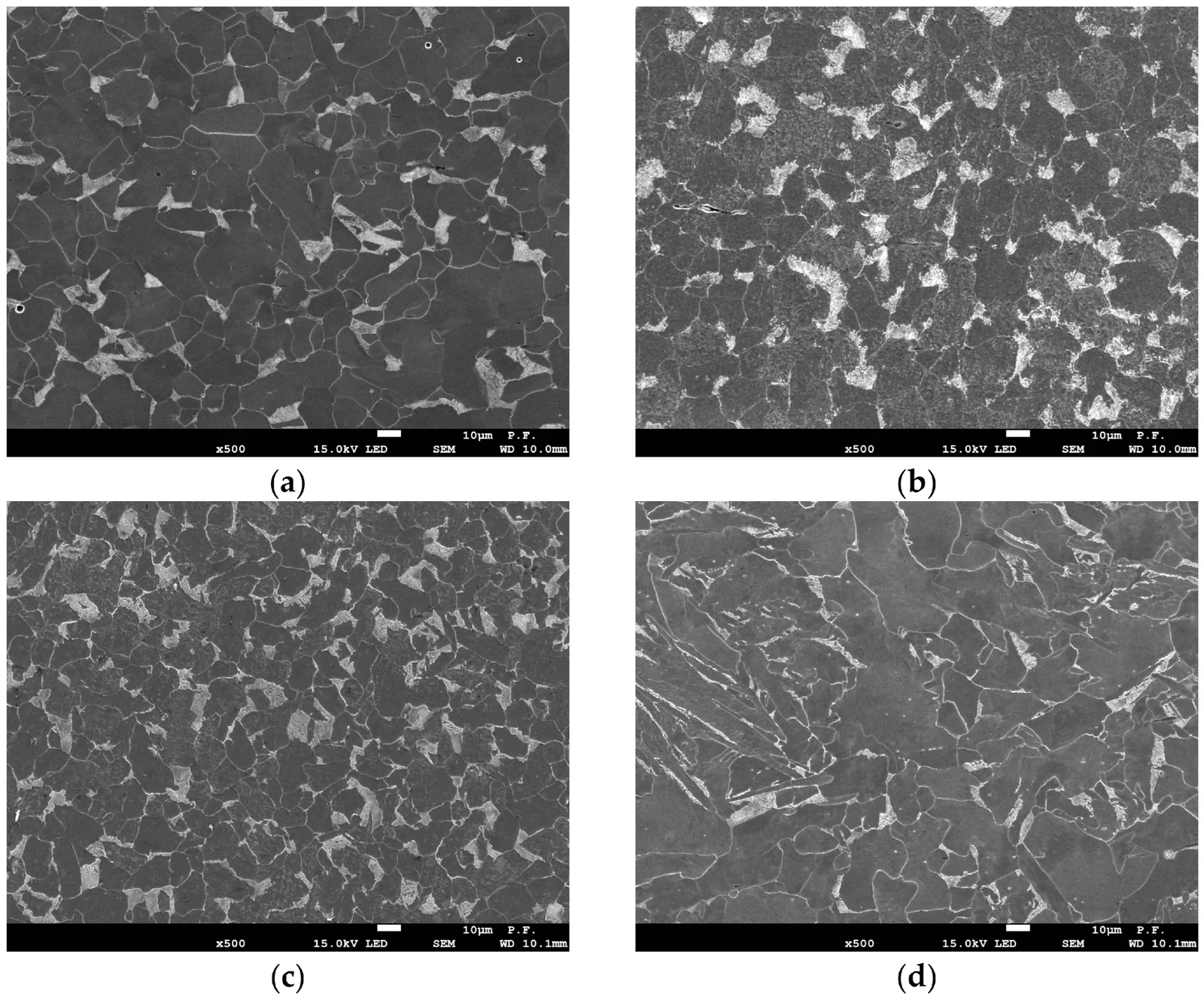
SEM micrographs of S235 structural steel etched with 4% nital after different thermal histories at 500× magnification: (**a**) as-received condition; (**b**) after exposure to 700 °C for 120 min; (**c**) after exposure to 900 °C for 120 min; (**d**) after exposure to 1100 °C for 120 min.

Microstructural observations were performed for the as-received steel and for representative specimens after thermal exposure at 700, 900, and 1100 °C for 120 min. These conditions were selected to provide a qualitative comparison of the microstructural state across the investigated temperature range at a constant exposure duration. Metallographic specimens were prepared by conventional grinding and polishing and subsequently etched with a 4% solution of nitric acid in ethanol (4% nital). Microstructural observations were performed using a JEOL JSM-7100F field-emission scanning electron microscope (SEM; JEOL Ltd., Akishima, Tokyo, Japan). Representative micrographs obtained at 500× magnification are presented in Figure 1.

### 2.2. Thermal Exposure

To obtain different thermally modified material conditions, the specimens were exposed to temperatures of 700, 900, and 1100 °C. For each temperature, exposure durations of 40, 80, and 120 min were investigated. Specimens maintained at room temperature without thermal treatment were used as the reference condition.

The selected temperatures were intended to represent progressively severe thermal exposures spanning conditions below, around, and above the temperature range in which substantial changes in the residual response of low-carbon structural steel may develop. The use of three widely separated temperature levels enabled the study to examine whether the subsequent AE response varied systematically with the severity of the preceding thermal exposure rather than focusing on a single post-fire condition.

Exposure durations of 40, 80, and 120 min were selected to introduce thermal-history variation at each nominal temperature while maintaining an identical temperature matrix. This design allowed the influence of exposure duration to be evaluated independently within each temperature level and enabled assessment of whether nominal maximum temperature alone was sufficient to describe the subsequent mechanical and AE response.

Thermal exposure was performed in a chamber furnace with a temperature-control accuracy of approximately ±5 °C. The specimens were placed in the furnace at ambient temperature and heated together with the furnace. The specimen-specific heating rate was not measured directly because no thermocouple was attached to the specimens. Consequently, the heating stage is defined by the furnace programme rather than by an experimentally determined specimen heating rate, and no precise heating-rate value is assigned to the material itself. Furnace temperature was monitored using a type-K thermocouple positioned in the central region of the chamber. The nominal exposure time was measured from the moment at which the furnace reached the target temperature. After completion of the prescribed exposure period, the specimens were removed from the furnace and cooled to ambient temperature in still laboratory air.

The reported exposure temperature represents the nominal furnace temperature rather than a direct measurement of the specimen temperature because no thermocouples were attached directly to the specimens. Consequently, the actual specimen-temperature history, including the time required for the specimens to reach the nominal furnace temperature, was not recorded. After cooling, the oxide scale was mechanically removed from the specimen surfaces. The cross-sectional dimensions used to calculate engineering stress were measured individually after thermal exposure and scale removal.

The complete experimental matrix used in the present study is summarized in Table 1. Three specimens were tested for each thermal condition.

### 2.3. Uniaxial Tensile Testing

Uniaxial tensile tests were performed at ambient temperature using a Zwick/Roell universal testing machine (ZwickRoell GmbH & Co. KG, Ulm, Germany) in accordance with ISO 6892-1 [25]. The force-measurement system satisfied the Class 1 accuracy requirements according to ISO 7500-1 [26], corresponding to the accuracy class specified for the verification of force-measuring systems used in uniaxial testing machines. The specimens were loaded continuously to fracture at a constant crosshead displacement rate of 5 mm/min. The crosshead displacement rate is reported as the machine-control parameter and should not be interpreted as the directly measured strain rate within the gauge section.

Force and crosshead displacement were recorded throughout each test. Strain within the gauge section was measured using a mechanical extensometer with an initial gauge length of 25 mm and a displacement resolution of approximately 1–2 μm. The tests were conducted at a laboratory temperature of 21 ± 1 °C and relative humidity of approximately 40–50%.

Engineering stress was calculated as:

(1) $$\sigma_{eng}=\frac{F}{A_{0}},$$

where *F* is the instantaneous tensile force and *A*_0_ is the initial measured cross-sectional area. In the present study, *A*_0_ denotes the cross-sectional area measured at the minimum-width section after thermal exposure, air cooling, and mechanical removal of the oxide scale, but before tensile testing.

Engineering strain was calculated as:

(2) $$\varepsilon_{eng}=\frac{\Delta L}{L_{0}},$$

where ΔL is the extensometer displacement and *L*_0_ is the initial gauge length.

Because the investigated S235 steel exhibited discontinuous yielding, both the upper yield strength, σ_YS,H_, and the lower yield strength, σ_YS,L_, were determined. The stage-resolved AE analysis was referenced to the lower yield load. Accordingly, *t_YS_* denotes the time corresponding to the lower yield point, whereas *t*_75YS_ denotes the time at which the applied force first reached 75% of the lower yield load.

Before the onset of necking, calculated true stress and true strain were obtained as:

(3) $$\sigma_{true}=\sigma_{eng}\times \left(1+\varepsilon_{eng}\right)$$

and

(4) $$\varepsilon_{true}=ln\left(1+\varepsilon_{eng}\right)$$

These relationships assume uniform deformation and volume constancy and were therefore not used to determine the local true stress after the onset of necking. The principal mechanical parameters considered in the analysis were the lower and upper yield strengths and the ultimate tensile strength.

The uncertainty associated with force measurement did not exceed the limits corresponding to class 1 of the testing system. Additional uncertainty arose from extensometer resolution and cross-sectional measurements. Because the same equipment and procedure were used for all conditions, the comparative analysis was performed under consistent measurement conditions.

### 2.4. Acoustic Emission Monitoring

Acoustic emission was recorded continuously during tensile testing using an AEWin acquisition system equipped with Express-8 acquisition cards [27]. Two Vallen VS75-SIC-40 dB sensors with integrated 40 dB preamplifiers and a nominal resonant frequency of 75 kHz were used for each specimen [28]. The sensors were positioned on opposite sides of the reduced section and mechanically secured to maintain stable contact throughout loading. Technical silicone was used as the coupling medium.

Before each test, sensor coupling and signal transmission were verified using the Hsu–Nielsen pencil-lead break procedure in accordance with the general approach described in ASTM E976 [29]. Pencil-lead breaks were performed at reproducible positions relative to the sensors. The test was used to confirm correct sensor operation and comparable coupling conditions rather than to provide absolute energy calibration.

The principal AE acquisition settings were as follows:

- Integrated preamplifier gain: 40 dB;
- Detection threshold: 40 dB;
- Sampling frequency: 10 MHz;
- Band-pass filter: 20–400 kHz;
- Peak definition time (PDT): 200 μs;
- Hit definition time (HDT): 800 μs;
- Hit lockout time (HLT): 1000 μs.

The detection threshold of 40 dB was maintained constant for all specimens to ensure identical acquisition conditions throughout the experimental programme. The threshold was selected to suppress low-level background and testing-system noise while retaining mechanically induced AE activity detectable during tensile loading. Its purpose was therefore to provide a consistent signal-detection criterion for comparative analysis rather than to define an intrinsic material-specific AE threshold.

Counts were defined as the number of threshold crossings within an individual AE hit. Cumulative counts represented the progressive sum of counts from the beginning of mechanical loading.

AE monitoring was performed using two sensors connected to two independent acquisition channels. The signals from the channels were not summed because the same source activity could be detected by both sensors, potentially resulting in duplicate counting. Channel 2 was selected before the comparative analysis and was used consistently for all specimens and thermal conditions. Channel 1 was retained as a control of sensor operation, signal transmission, and coupling stability. Therefore, the two channels were recorded independently, but only channel 2 was used to calculate the stage-resolved AE descriptors reported in this study. Because the use of a single channel may influence the absolute cumulative-count values, the results are interpreted comparatively within the fixed sensor arrangement and acquisition configuration.

Because AE counts depend on the threshold, hit-definition parameters, sensor response, coupling, specimen geometry, and propagation conditions, they were interpreted as configuration-dependent comparative descriptors rather than intrinsic material properties. All specimens were tested using the same acquisition configuration.

The analysis intentionally focused on counts and cumulative counts. This choice was consistent with the principal objective of evaluating the stage-dependent accumulation of detectable AE activity relative to characteristic mechanical points. The study did not aim to identify individual source mechanisms or classify damage modes, which would require complementary waveform-, energy-, amplitude-, and frequency-based descriptors. Consequently, the results were interpreted comparatively within the fixed acquisition configuration used for all specimens.

**Figure 2:**
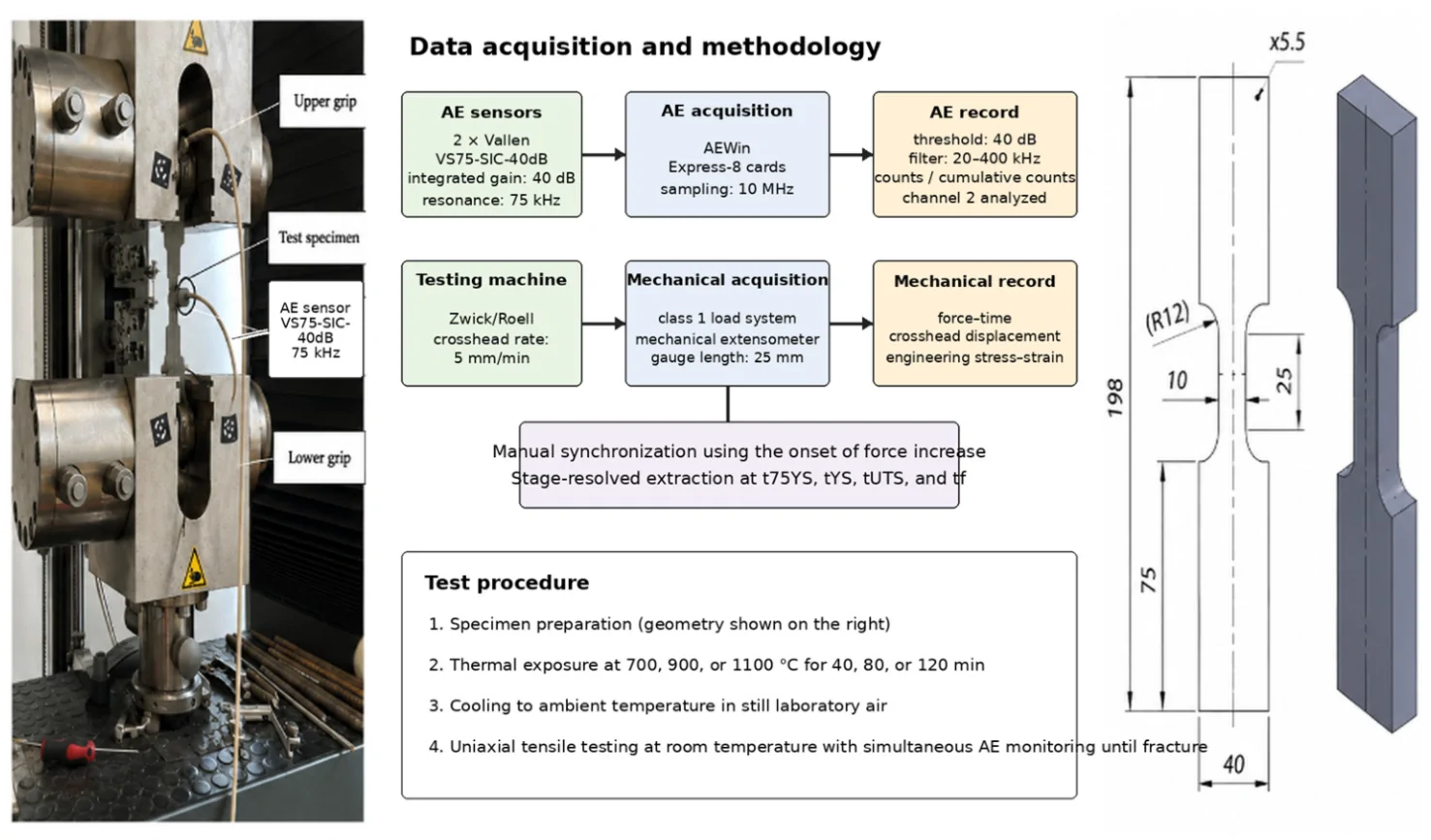
Experimental setup and schematic overview of the methodology used for tensile testing and acoustic emission monitoring of thermally exposed S235 steel specimens. Specimen dimensions are given in millimetres.

The overall experimental procedure, including specimen geometry, thermal exposure, tensile testing, and simultaneous acoustic emission monitoring, is summarized in Figure 2.

### 2.5. Synchronization of Mechanical and Acoustic Emission Data

The mechanical and AE datasets were exported to Microsoft Excel and arranged on a common time axis. A constant time offset was applied by aligning the onset of AE acquisition with the beginning of the increase in the force–time record. The synchronized records were subsequently matched at a temporal resolution of 1 s. Consequently, the characteristic mechanical times and the corresponding cumulative-count values were subject to a synchronization uncertainty of approximately ±1 s. This uncertainty is most relevant close to stages characterized by a rapid accumulation of AE counts, particularly around yielding, because a small temporal displacement may change the exact cumulative value assigned to the mechanical marker. It is less consequential during intervals in which the cumulative-count curve changes slowly. Accordingly, the values extracted at the characteristic times should be interpreted as mechanically referenced values with an approximately ± 1 s temporal uncertainty rather than exact instantaneous AE quantities. No local stretching, nonlinear time transformation, or stage-dependent adjustment of either record was performed.

Four characteristic mechanical times were identified separately for each specimen:

- *t*_75YS_: the first time at which the applied force reached 75% of the lower yield load;
- *t*_YS_: the time corresponding to the lower yield point;
- *t*_UTS_: the time corresponding to the maximum engineering stress;
- *t*_f_: the time of final fracture.

Cumulative AE counts were then extracted at these mechanically defined times. The values obtained for the three specimens within each thermal condition were summarized using the median because of the limited sample size and the potentially non-normal distribution of AE quantities.

### 2.6. Stage-Resolved Analysis of AE Activity

The analysis was designed to compare the evolution of cumulative AE activity with conventional residual mechanical characteristics. Instead of considering only the total cumulative counts recorded up to fracture, AE activity was evaluated at the mechanically defined stages *t*_75YS_, *t*_YS_, *t*_UTS_, and *t*_f_.

The cumulative-count parameters were defined as:

(5) $$N_{cum,75YS}=N_{\left(cum\right)\left(t_{75YS}\right)}$$

(6) $$N_{cum,YS}=N_{\left(cum\right)\left(t_{YS}\right)}$$

(7) $$N_{cum,UTS}=N_{\left(cum\right)\left(t_{UTS}\right)}$$

(8) $$N_{cum,f}=N_{\left(cum\right)\left(t_{f}\right)}.$$

To describe the proportion of total AE activity accumulated by the individual mechanical stages, the following normalized ratios were calculated:

(9) $$R_{75YS}=\frac{N_{cum,75YS}}{N_{cum,f}},$$

(10) $$R_{YS}=\frac{N_{cum,YS}}{N_{cum,f}},$$

(11) $$R_{UTS}=\frac{N_{cum,UTS}}{N_{cum,f}}.$$

For the condition-level comparisons, the normalized ratios were calculated from the condition-level median cumulative counts.

Relative changes with respect to the reference condition were calculated as:

(12) $$\Delta X=\frac{X_{i}-X_{0}}{X_{0}}\times 100\%$$

where *X*_i_ and *X*_0_ are the aggregated values for the thermally exposed and as-received conditions, respectively. Relative changes in mechanical properties were calculated from the arithmetic means, whereas relative changes in AE descriptors were calculated from the corresponding medians.

The 75%F*_YS_* level was selected as a normalized pre-yield reference point sufficiently separated from the macroscopic yield transition. Because this level was determined retrospectively from the lower yield load of each specimen, *N*_cum,75YS_ was interpreted as a stage-resolved comparative descriptor rather than as an independently applicable prospective diagnostic threshold.

Three specimens were tested for each condition. Individual values, medians, and minimum–maximum ranges were used to describe within-condition variability. Given the limited sample size, the analysis was treated as exploratory and comparative. The reported ranges were not interpreted as formal evidence of statistical separation between conditions, and no universal classification thresholds were derived.

To examine whether the normalized stage ratios were systematically associated with the magnitude of total AE activity, Spearman rank correlations were additionally calculated between the condition-level median *N*_cum,f_ and the corresponding *R*_75YS_, *R*_YS_, and *R*_UTS_ values.

## 3. Results

### 3.1. Initial Microstructure and Residual Mechanical Properties

The as-received S235 steel exhibited a conventional ferritic–pearlitic microstructure, with predominantly equiaxed ferrite grains and pearlite regions distributed mainly along and between the ferrite grains (Figure 1a). Thermal exposure produced visible changes in the microstructural appearance. After exposure to 700 °C for 120 min (Figure 1b), the ferritic–pearlitic character remained distinguishable, although differences in the morphology and distribution of the microstructural constituents were observed relative to the as-received condition. More pronounced changes were visible after exposure to 900 °C for 120 min (Figure 1c), while the specimen exposed to 1100 °C for 120 min (Figure 1d) exhibited the most distinctly modified microstructural appearance among the representative conditions shown.

These observations provide qualitative evidence that the investigated thermal histories modified the microstructural state of the steel. However, because the present study did not include quantitative metallographic measurements or phase identification, the micrographs are used only for qualitative comparison and are not employed to assign specific microstructural mechanisms to the observed changes in AE activity. A systematic quantitative microstructural investigation across all temperature–duration combinations remains outside the scope of the present study.

**Figure 3:**
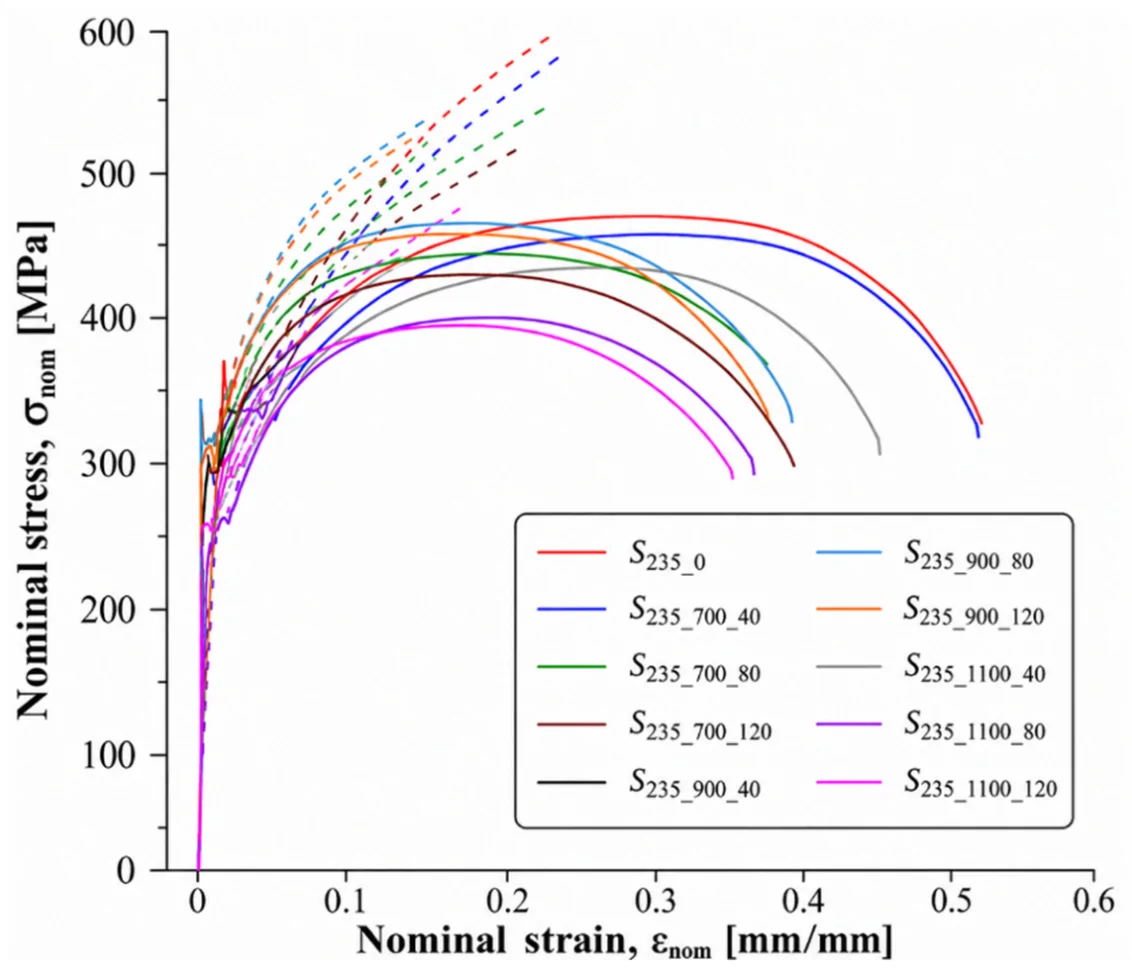
Engineering and calculated true stress–strain curves of representative S235 steel specimens under different thermal exposure conditions. For each condition, the specimen whose lower yield strength was closest to the series mean was selected. Solid lines represent engineering stress–strain curves, whereas dashed lines represent calculated true stress–strain curves up to the onset of necking.

The residual mechanical response of S235 steel was affected by both the temperature and duration of thermal exposure. Figure 3 presents the representative nominal and true stress–strain curves obtained for the investigated thermal conditions. Differences between the series were observed in the yield region, maximum stress, and subsequent deformation response. The characteristic mechanical points identified from the stress–strain response were subsequently used as reference stages for the stage-resolved AE analysis.

Table 2 summarizes the nominal lower and upper yield strengths and ultimate tensile strength obtained for the investigated conditions. The values are presented as the mean ± standard deviation calculated from three specimens.

Values are reported as mean ± standard deviation for three specimens. The upper yield strength was not identified for the S235_1100_80 series because a distinct upper-yield point was not observed in the corresponding stress–strain responses.

Thermal exposure produced a non-monotonic response with respect to temperature and exposure duration. For the reference condition, the mean nominal lower yield strength, σ_YS,L_, was 324.14 MPa and the ultimate tensile strength, σ_UTS_, was 463.06 MPa. Exposure to 700 °C for 40 min resulted in only moderate reductions in these parameters, to 317.67 and 447.62 MPa, respectively. Increasing the exposure duration at 700 °C progressively reduced both properties, with σ_YS,L_ decreasing to 272.50 MPa and σ_UTS_ to 418.48 MPa after 120 min.

A different response was observed at 900 °C. After 40 min, the mean σ_YS,L_ and σ_UTS_ values were 326.61 and 458.85 MPa, respectively, and therefore remained close to the reference values. Longer exposure resulted in a progressive decrease in yield strength, whereas the reduction in ultimate tensile strength remained comparatively limited. After 120 min at 900 °C, σ_YS,L_ decreased to 279.81 MPa, while σ_UTS_ remained at 450.45 MPa.

The greatest deterioration of the residual mechanical properties was generally observed following exposure to 1100 °C. The mean lower yield strength decreased to 266.18 MPa after 40 min and to approximately 232 MPa after 80–120 min. These results demonstrate that the magnitude of the residual mechanical changes depended on the specific temperature–duration combination and provide the mechanical reference against which the sensitivity of the AE response was subsequently evaluated.

### 3.2. Evolution of Cumulative AE Activity During Tensile Loading

**Figure 4:**
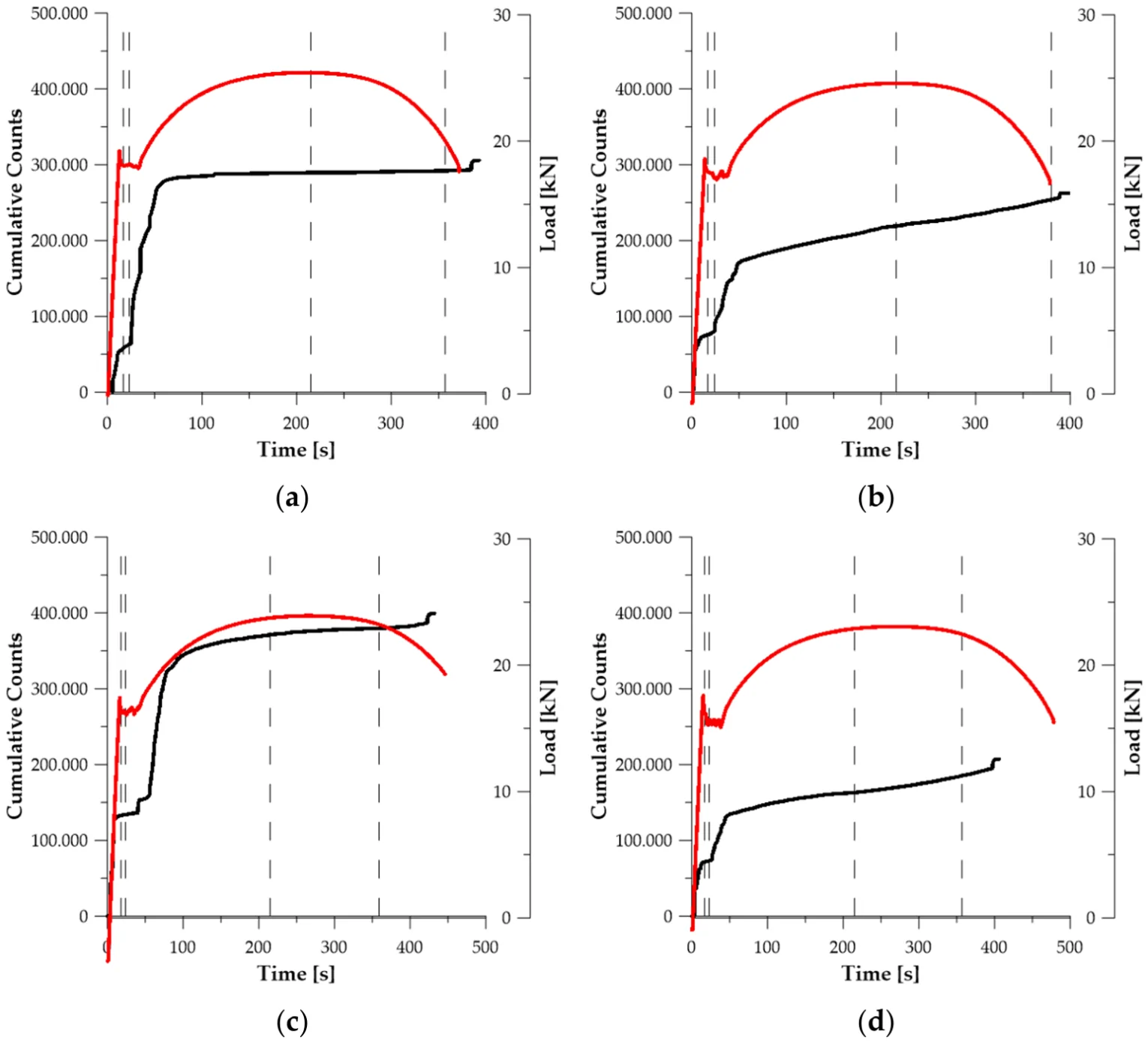

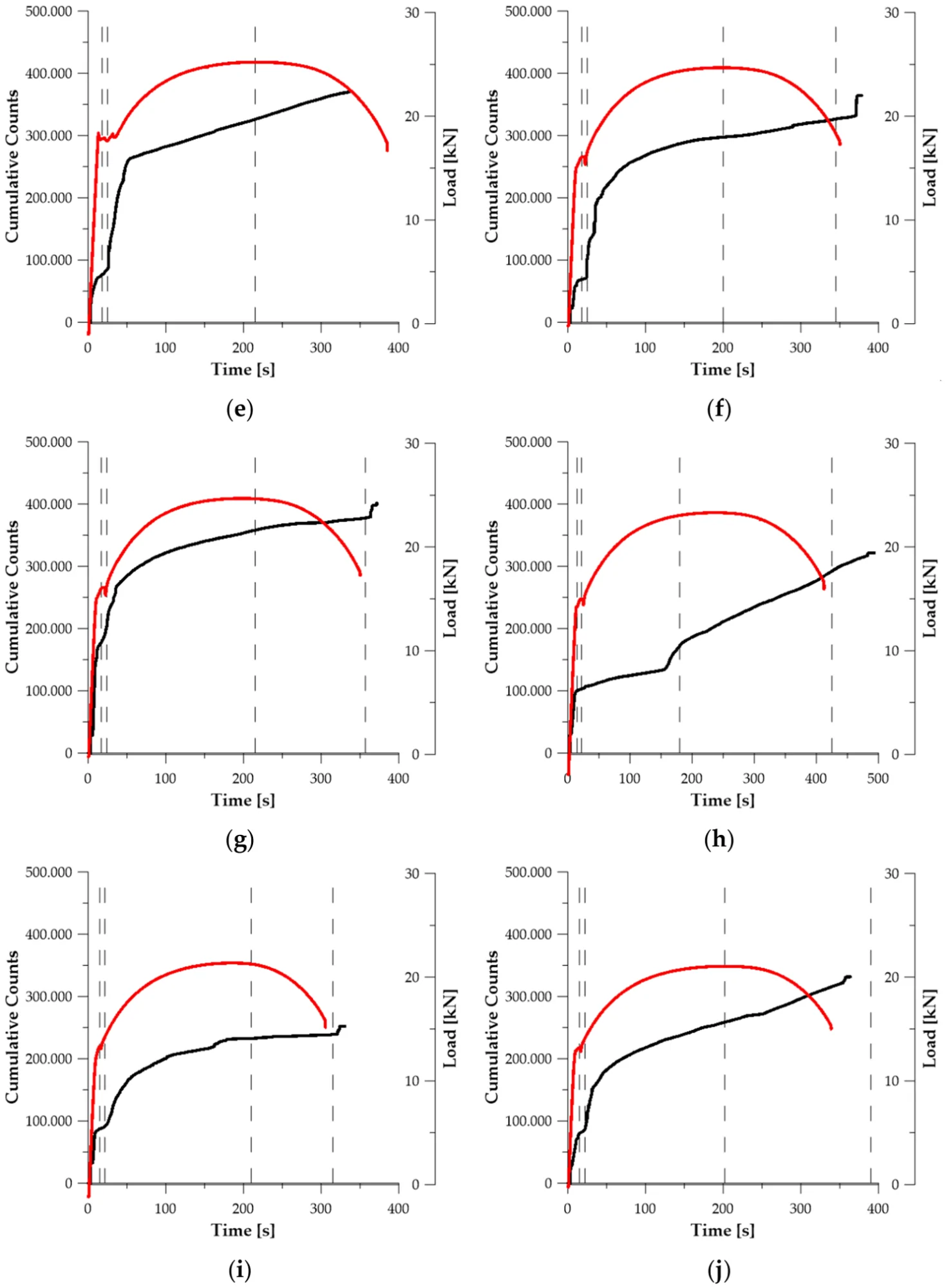
Evolution of cumulative acoustic emission counts and load as a function of test time for representative S235 steel specimens: (**a**) S235_0; (**b**) S235_700_40; (**c**) S235_700_80; (**d**) S235_700_120; (**e**) S235_900_40; (**f**) S235_900_80; (**g**) S235_900_120; (**h**) S235_1100_40; (**i**) S235_1100_80; and (**j**) S235_1100_120. The characteristic times *t*_75YS_, *t*_YS_, *t*_UTS_, and *t*_f_ indicate 75% of the lower yield load, the lower yield point, maximum load, and fracture, respectively. The black curves represent cumulative AE counts recorded by channel 2, whereas the red curves represent tensile load. For each condition, the specimen with *N*_cum,75YS_ closest to the series median was selected as representative.

Figure 4 compares the evolution of cumulative AE counts with the corresponding load–time response for representative specimens from each investigated condition. Four mechanically defined characteristic times were used to relate changes in acoustic activity directly to the tensile response: *t*_75YS_, corresponding to 75% of the lower yield load; *t*_YS_, corresponding to the lower yield point; *t*_UTS_, corresponding to the maximum load and ultimate tensile strength; and *t*_f_, corresponding to final fracture.

Pronounced differences between the cumulative-count histories were observed despite comparatively limited differences in the mechanical response for some thermal conditions. The differences concerned not only the final number of accumulated counts but also their distribution over the successive stages of tensile deformation. In several thermally exposed conditions, enhanced accumulation of AE counts occurred already during the pre-yield and yielding stages, whereas subsequent accumulation between yielding, ultimate tensile strength, and fracture was comparatively less pronounced. This behaviour indicates that evaluation of total cumulative counts alone may obscure changes in the stage of deformation at which AE activity is generated.

### 3.3. Cumulative AE Activity at Characteristic Mechanical Stages

The median cumulative AE counts evaluated at the characteristic mechanical stages showed substantial descriptive differences between the reference and thermally exposed conditions (Table 3). For the reference material, the median cumulative counts were 57,552 at *t*_75YS_ and 63,142 at *t*_YS_. Depending on the thermal condition, *N*_cum,75YS_ ranged from 47,184 to 178,636, while *N*_cum,YS_ ranged from 48,285 to 203,479. The corresponding ranges at UTS and fracture were 162,872–357,790 and 185,165–377,598 counts, respectively.

Particularly pronounced differences were observed during the early stages of loading. After exposure to 900 °C for 120 min, *N*_cum,75YS_ reached 178,636 counts, compared with 57,552 counts in the reference condition, corresponding to an increase of 210.4%. At yielding, the difference was even slightly greater, with *N*_cum,YS_ increasing from 63,142 to 203,479 counts (+222.3%). In contrast, the differences relative to the reference condition were considerably smaller at UTS (+23.6%) and fracture (+29.1%).

The 1100 °C/40 min condition provides a further example of the importance of evaluating the temporal distribution of AE activity. The median cumulative counts at fracture were 292,180, almost identical to the reference value of 292,460. Nevertheless, *N*_cum,75YS_ increased from 57,552 to 95,022 (+65.1%), while *N*_cum,YS_ increased from 63,142 to 102,911 (+63.0%). Conversely, the cumulative activity at UTS was 40.5% lower than in the reference condition. Thus, similar final cumulative AE activity did not imply a similar evolution of acoustic activity during deformation.

The response was not monotonic for all thermal conditions. For example, exposure to 700 °C for 80 min resulted in lower cumulative counts than the reference condition at all four characteristic stages, whereas exposure to 900 °C for 120 min produced the largest increase in early AE activity. These results indicate that the cumulative AE response depended on the specific combination of exposure temperature and duration rather than varying monotonically with temperature alone.

Because AE counts are configuration-dependent quantities, the numerical values reported in Table 3 should be interpreted comparatively within the acquisition configuration used in this study.

### 3.4. Relative Changes in Stage-Resolved Cumulative AE Activity

To compare the sensitivity of AE activity to thermal exposure at successive stages of tensile deformation, the median cumulative counts for each thermal condition were expressed as relative changes with respect to the reference S235_0 condition. This normalization enables direct comparison of both the magnitude and direction of changes in AE activity at the pre-yield, yield, UTS, and fracture stages.

**Figure 5:**
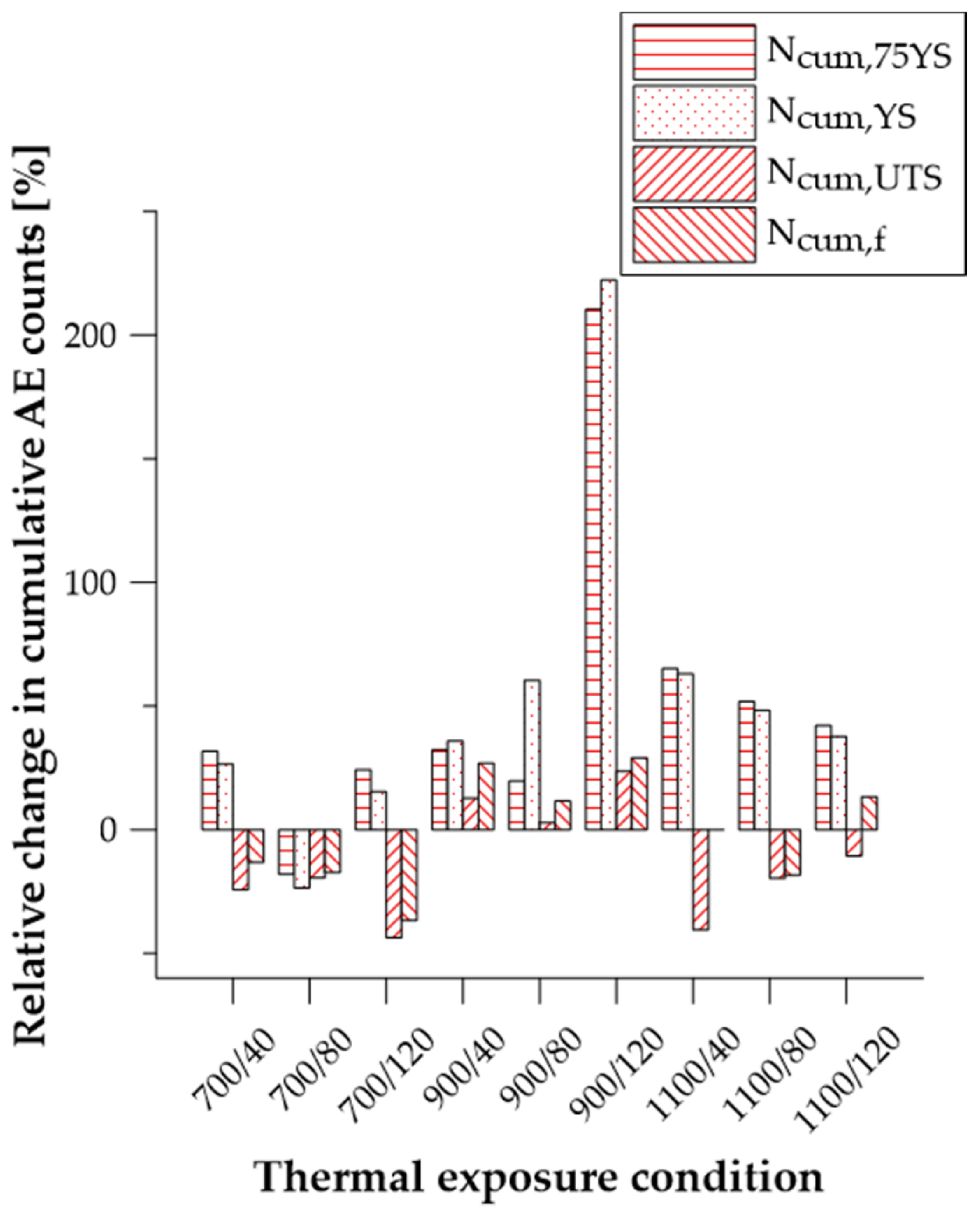
Relative changes in median cumulative AE counts recorded by channel 2 at characteristic stages of tensile deformation with respect to the reference S235_0 condition (*n* = 3 per condition). Positive values indicate an increase and negative values a decrease relative to the reference material.

Figure 5 emphasizes that the magnitude and direction of the differences in AE activity depended strongly on the stage of tensile deformation. The greatest relative differences were generally observed at *t*_75YS_ and *t_YS_*, particularly for the 900 °C/120 min and 1100 °C conditions. Importantly, these early differences did not necessarily persist until fracture. For example, the 1100 °C/40 min condition exhibited increases of 65.1% and 63.0% at *t*_75YS_ and *t_YS_*, respectively, while the difference in final cumulative counts was negligible (−0.1%). This convergence of total AE activity despite markedly different early responses indicates that stage-dependent differences may be obscured when only the final cumulative value is considered.

To assess the repeatability of the mechanically referenced pre-yield AE response, the dispersion of cumulative counts across the three replicate specimens was evaluated at 75% of the lower yield load. Table 4 presents the individual values, medians, and minimum–maximum ranges for *N*_cum,75YS_.

The relative range was calculated as:

(13) $$\frac{N_{max}-N_{min}}{N_{median}}\times 100\%,$$

The within-series ranges provide a descriptive indication of specimen-to-specimen variability in the early AE response. Owing to the limited number of specimens, these ranges were not interpreted as formal evidence of statistical separation between thermal conditions. The subsequent comparisons use median values to reduce the influence of individual extreme responses.

### 3.5. Redistribution of AE Activity over the Tensile-Loading History

**Figure 6:**
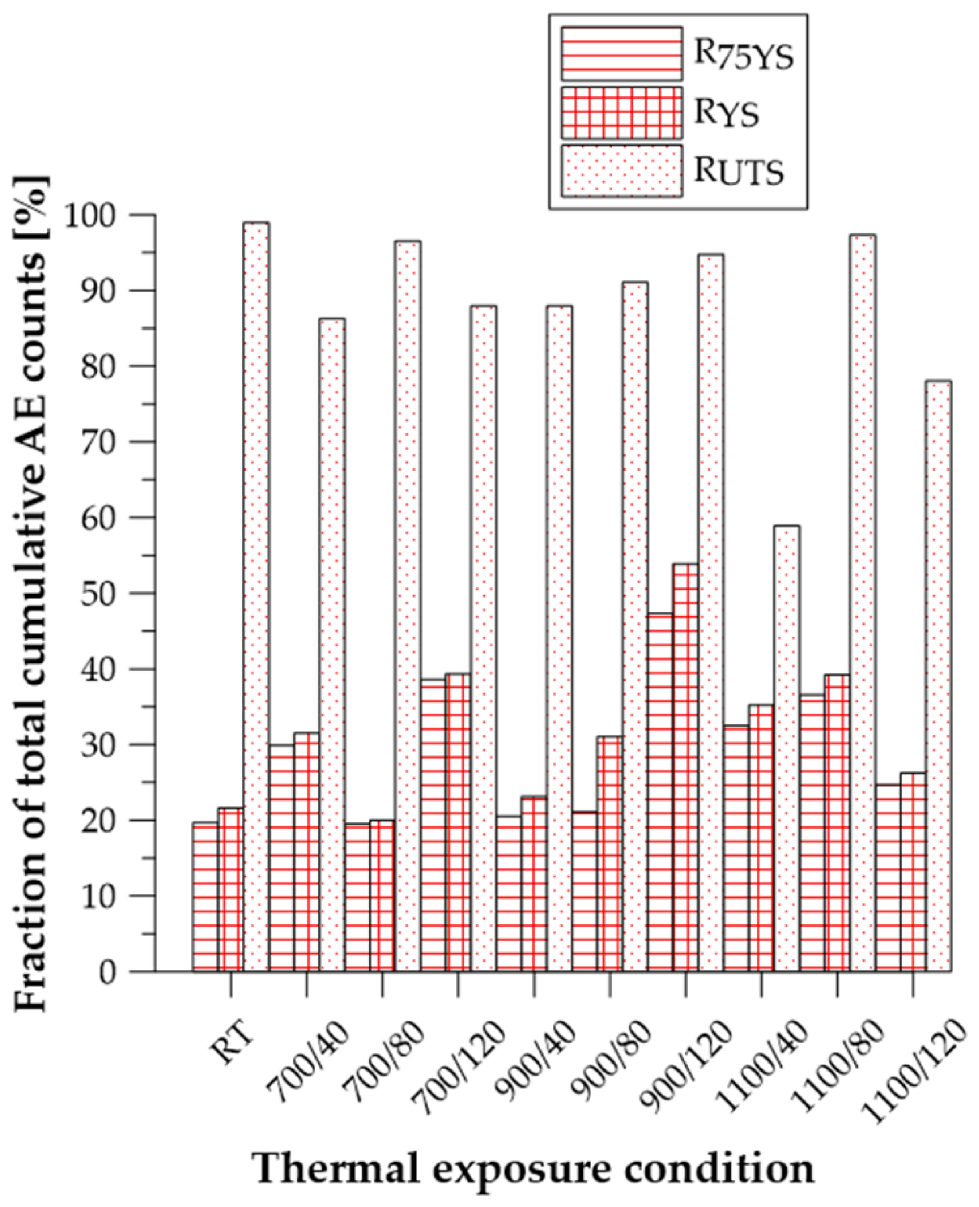
Fractions of total cumulative AE counts recorded by channel 2, calculated from the condition-level median cumulative counts reported in Table 3, and accumulated at 75% of the lower yield load (*R*_75YS_), at the lower yield point (*R*_YS_), and at ultimate tensile strength (*R*_UTS_) for the investigated thermal exposure conditions (median counts based on *n* = 3 specimens per condition).

To determine whether thermal exposure altered the timing of AE activity rather than only its final magnitude, cumulative counts recorded at 75% of the lower yield load, at the lower yield point, and at UTS were normalized by the total cumulative counts recorded up to fracture. The resulting ratios, *R*_75YS_, *R*_YS_, and *R*_UTS_, describe the fractions of the total AE activity accumulated by successive mechanical stages (Figure 6).

In the reference condition, 19.7% of the total cumulative counts were generated by the time the force reached 75% of the lower yield load, and 21.6% had accumulated by the lower yield point. Approximately 99.0% of the final counts had accumulated by UTS. Thus, only approximately one fifth of the total activity occurred before yielding, whereas little additional activity was recorded after the maximum load.

The strongest redistribution towards the pre-yield stage was observed after exposure to 900 °C for 120 min. For this condition, *R*_75YS_ and *R*_YS_ reached 47.3% and 53.9%, respectively. Elevated pre-yield contributions were also observed after exposure to 700 °C for 120 min and after selected 1100 °C treatments.

A different distribution was observed for the 1100 °C/40 min condition. Although *R*_75YS_ and *R*_YS_ reached 32.5% and 35.2%, respectively, *R*_UTS_ was only approximately 58.9%. Consequently, approximately 41% of the final cumulative counts were generated between UTS and fracture, compared with approximately 1% in the reference condition.

Because the normalized ratios contain the final cumulative count in the denominator, their potential dependence on the total recorded AE activity was additionally examined across the ten investigated thermal conditions. Spearman rank correlations between the median final cumulative counts, *N*_cum,f_, and *R*_75YS_, *R*_YS_, and *R*_UTS_ were −0.103 (*p* = 0.777), −0.115 (*p* = 0.751), and −0.176 (*p* = 0.627), respectively. Thus, no statistically significant monotonic association between the total cumulative-count magnitude and any of the three normalized ratios was identified at the condition level. This result indicates that the observed differences in *R* primarily describe redistribution of recorded AE activity over the tensile-loading history rather than a systematic drift associated with increasing or decreasing final cumulative counts.

These findings demonstrate that similar final cumulative AE activity may result from markedly different distributions of activity over the tensile-loading history.

### 3.6. Comparison of Relative Changes in Pre-Yield AE Activity and Lower Yield Strength

**Figure 7:**
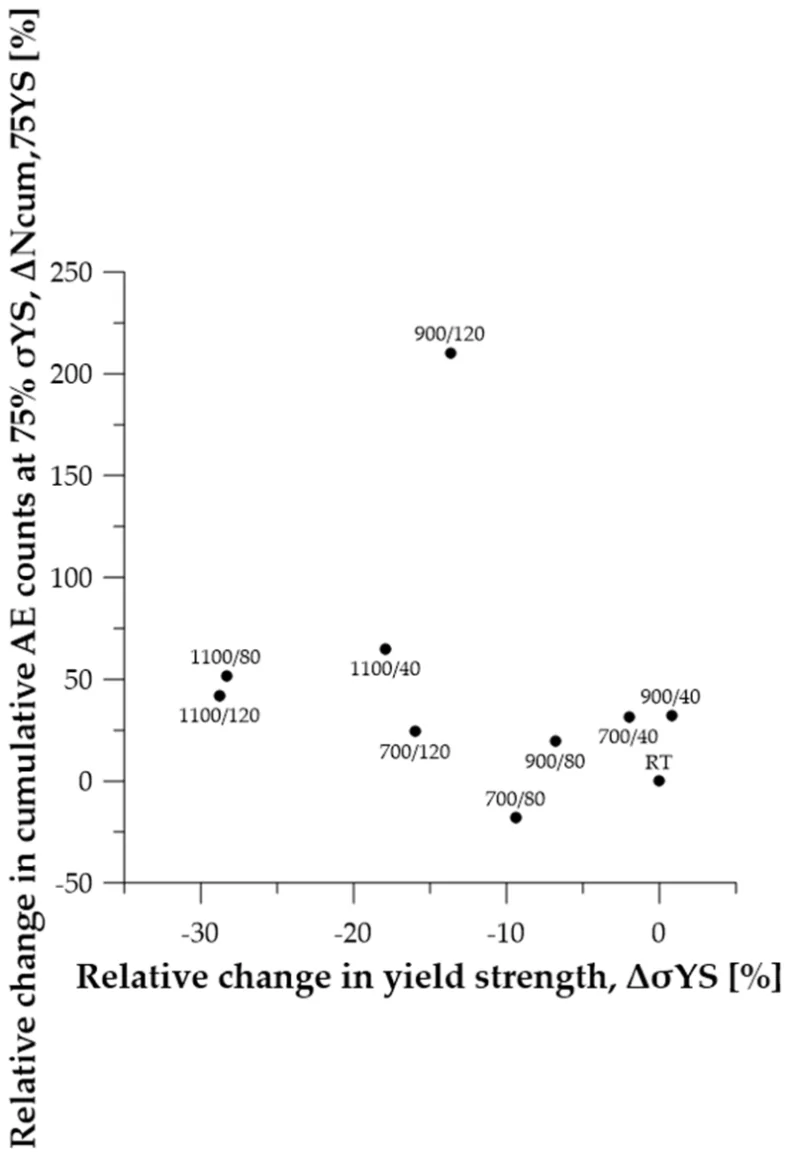
Relationship between the relative change in mean lower yield strength, Δσ_YS,L_, and the relative change in median cumulative AE counts recorded by channel 2 at 75% of the lower yield load, Δ*N*_cum,75YS_, with respect to the reference S235_0 condition. Labels indicate the nominal thermal exposure conditions.

Figure 7 compares the relative change in mean lower yield strength, Δσ_YS,L_, with the corresponding relative change in median cumulative AE counts recorded at 75% of the lower yield load, Δ*N*_cum,75YS_, both expressed relative to the reference condition. The comparison reveals that changes in early AE activity were considerably greater than the corresponding changes in residual yield strength for several thermal exposure conditions.

The most pronounced response was observed after exposure to 900 °C for 120 min. Although the mean lower yield strength decreased by approximately 14%, the median cumulative AE counts recorded at 75% of the lower yield load increased by approximately 210% relative to the reference condition. A similar, although less pronounced, divergence between the mechanical and acoustic responses was observed for several other thermally exposed conditions.

Importantly, the relationship between the change in yield strength and the change in early cumulative AE activity was not monotonic. Comparable reductions in yield strength could be accompanied by markedly different changes in *N*_cum,75YS_, while conditions exhibiting relatively small mechanical changes could produce substantial increases in early AE activity. This indicates that the relative changes in the AE descriptor were numerically greater than the corresponding relative changes in residual lower yield strength.

The comparison shows that *N*_cum,75YS_ did not simply reproduce the magnitude of residual strength loss. Instead, the mechanically referenced AE parameter exhibited a pattern that was not proportional to the corresponding change in lower yield strength, indicating that it provided complementary information on the pre-yield deformation history. The observed 210% increase should be interpreted as a large relative change in a configuration-dependent AE descriptor, rather than as direct evidence that AE is quantitatively 15 times more diagnostically sensitive than yield strength.

## 4. Discussion

The present results indicate that the diagnostic value of acoustic emission in thermally exposed S235 steel is determined not only by the overall amount of recorded activity but, importantly, by when this activity develops relative to the mechanical response. Conventional tensile parameters describe selected macroscopic characteristics of the material, whereas AE records transient processes occurring throughout deformation. Consequently, thermal modification of the initial material state may affect the AE response before substantial differences become apparent in selected residual strength parameters.

### 4.1. Significance of the Early AE Response

The mechanically referenced analysis provides a different perspective from approaches based predominantly on the total AE activity accumulated up to failure. In the present study, cumulative counts were evaluated at characteristic stages defined from the tensile response, including a pre-yield level, yielding, UTS, and fracture. This approach makes it possible to distinguish a change in the timing of acoustic activity from a simple increase or decrease in its final magnitude.

The enhanced contribution of AE before macroscopic yielding observed for selected thermal histories suggests that thermal exposure modified the processes activated during the transition from nominally elastic behaviour towards plastic deformation. AE generated during deformation of metallic materials has been associated with processes including dislocation motion and multiplication, localized plastic deformation, and the initiation and development of microstructural damage [12,16,19]. Accordingly, changes in the pre-yield AE response may reflect thermally induced modification of the initial microstructural and stress state from which subsequent deformation develops.

This interpretation should nevertheless be treated with appropriate caution. The present measurements do not permit individual AE signals to be assigned directly to specific microstructural mechanisms. Accordingly, the physical interpretation proposed here is phenomenological: the AE results demonstrate modification of the evolution of irreversible processes during loading, but they do not identify the underlying microstructural source of that modification. Therefore, the observed changes in early AE activity cannot by themselves demonstrate that a particular deformation or damage mechanism was dominant. Rather, they indicate that thermal exposure altered the evolution of irreversible processes capable of generating detectable acoustic activity during the approach to macroscopic yielding.

From this perspective, the AE response should not be regarded as a direct measure of residual yield strength. Instead, it provides complementary information on the evolution of deformation processes leading to macroscopic yielding. This distinction is particularly relevant diagnostically because a change in a mechanically referenced pre-yield AE descriptor may become apparent while the corresponding change in residual strength remains comparatively limited. The comparison between mechanical and acoustic responses therefore supports interpretation of early AE evolution as a sensitive descriptor of thermally modified material behaviour rather than as a surrogate measurement of strength itself.

It should be emphasized that *N*_cum,75YS_ is retrospective in the present experimental framework because determination of the corresponding force level requires prior identification of the lower yield load. The parameter therefore enables normalized comparison of pre-yield AE histories but does not yet constitute an independently applicable field-diagnostic threshold. A prospective criterion would require evaluation at an externally defined common stress, strain, or service-load level.

The use of cumulative counts provides a deliberately simple and transparent description of the timing of AE activity. Its principal advantage is that it enables direct comparison of the fraction of detected acoustic activity accumulated before yielding, at yielding, at UTS, and up to fracture. The descriptor is therefore suitable for addressing the central question of the present study—whether thermal exposure redistributes detectable AE activity over the tensile-loading history.

### 4.2. Redistribution Rather than Simple Amplification of AE Activity

An important implication of the present results is that thermal exposure cannot be described simply as producing either more or less AE activity. Instead, the acoustic activity appears to be redistributed over the tensile-loading history. This interpretation explains why specimens with comparable total cumulative counts at fracture may nevertheless exhibit substantially different AE histories.

This finding has methodological consequences. Total counts recorded at fracture integrate acoustic activity originating from different stages of deformation and consequently contain limited information about when the underlying AE-generating processes were activated. Similar final cumulative values may therefore result from different loading histories: one characterized by a relatively large contribution of early activity and another by greater activity during advanced plastic deformation, localization, or the post-UTS stage.

Normalization with respect to the total cumulative activity preserves information on this temporal distribution and enables the contribution of mechanically defined stages to be compared between thermal histories. In this context, the cumulative AE response is more appropriately considered as a deformation-history descriptor than solely as an accumulated damage quantity.

The differences observed in the contribution of post-UTS activity further support this interpretation. After the maximum load is reached, deformation becomes increasingly localized and progresses towards necking and final fracture. Changes in the proportion of AE generated during this stage therefore suggest that thermal history affects not only early deformation but also the distribution of acoustic activity during localization and failure. Consequently, reducing the entire AE record to a single cumulative value at fracture may conceal diagnostically relevant differences in the evolution of the material response.

Counts are additionally influenced by waveform duration and frequency content because a longer oscillatory hit may generate more threshold crossings than a shorter hit, even if both originate from a similar physical process. Confirmation using complementary descriptors such as hit number, signal energy, amplitude, or duration would therefore strengthen the interpretation of the observed redistribution.

### 4.3. Effect of Thermal History

Metallographic observations confirmed the ferritic–pearlitic microstructure of the as-received S235 steel and revealed visible changes in microstructural appearance after representative thermal exposures at 700, 900, and 1100 °C for 120 min (Figure 1). These qualitative observations support the conclusion that the thermal history modified the material state before subsequent tensile loading. However, because quantitative metallographic measurements and phase identification were not performed, the observed differences in AE activity cannot be attributed directly to specific changes in grain size, phase morphology, dislocation structure, or residual stresses. Such mechanisms are therefore discussed only as physically plausible factors contributing to the thermally modified mechanical and acoustic response and require dedicated quantitative verification.

The absence of a simple monotonic relationship between nominal furnace temperature and AE response is consistent with the combined influence of exposure duration and subsequent cooling conditions. Exposure duration determines the time available for thermally activated processes, whereas the subsequent cooling path influences the residual condition from which tensile deformation begins. Temperature, exposure duration, and cooling conditions therefore determine the extent to which thermally activated processes can develop before subsequent mechanical loading. This explanation is mechanistically plausible but should not be treated as direct evidence of specific microstructural transformations [5,6,8,9].

For low-carbon structural steel, heating and subsequent cooling may modify several interacting characteristics of the material, including the dislocation structure, residual stress state, grain-scale deformation behaviour and, depending on the severity of thermal exposure, the microstructural state. More generally, recent investigations of metallic materials have demonstrated that internal stress and obstacles to dislocation motion can substantially influence the subsequent deformation response [30]. Such modifications do not necessarily produce proportional changes in yield strength, ultimate tensile strength, and acoustic activity.

A one-to-one relationship between an individual AE descriptor and a single mechanical property should therefore not be expected. The lack of such a simple relationship in the present results does not diminish the diagnostic value of AE; rather, it indicates that the acoustic response contains information that is at least partly independent of conventional residual-strength parameters.

Exposure duration is particularly relevant in this context. It should not be considered merely as a secondary parameter accompanying peak temperature because it determines the time available for thermally activated processes to develop. Different temperature–duration combinations may therefore produce different initial states for subsequent deformation even when their residual macroscopic mechanical characteristics are relatively similar. This provides a plausible explanation for the observed dependence of the AE evolution on the combined thermal exposure conditions rather than on temperature alone.

### 4.4. Implications for AE-Based Assessment of Fire-Exposed Steel

From the perspective of structural diagnostics, the principal outcome of the present study is that mechanically referenced AE descriptors may provide information that is not available from either residual strength parameters or final cumulative AE activity considered independently. The proposed approach does not require the absolute number of AE counts to be interpreted as a universal material property. Instead, acoustic activity is evaluated relative to reproducible stages of the mechanical response.

This distinction is important because absolute AE quantities depend on several experimental and measurement-related factors, including acquisition threshold, sensor coupling, specimen geometry, wave propagation conditions, sensor characteristics, and data-acquisition settings. These factors can affect the recorded number and characteristics of AE signals and complicate direct comparison of absolute values between different experimental configurations [15,31]. The present analysis was therefore intentionally comparative, with emphasis on consistent differences between thermal conditions tested using the same experimental configuration.

Ratios describing the fraction of total activity accumulated at mechanically defined stages may partly reduce the importance of differences in absolute signal quantity while preserving information on the temporal distribution of acoustic activity. They should not, however, be regarded as completely independent of the measurement configuration. Their principal advantage in the present context is that they provide a normalized description of how the recorded acoustic activity develops over the mechanical-loading history.

The pre-yield region is especially attractive from a diagnostic perspective. If changes in AE behaviour can be identified before macroscopic yielding, the acoustic response may provide information on thermally modified material behaviour before extensive plastic deformation and failure occur. Previous investigations of metallic materials have also demonstrated that AE activity can develop before or around macroscopic yielding and that its evolution can be associated with changes in the underlying deformation processes [12,16,19].

The present experiments do not establish a nondestructive field-assessment procedure. The proposed mechanically referenced levels were defined retrospectively from tensile tests and require loading of a material specimen to a known fraction of its lower yield load. Application to an existing structural member would require an independent estimate of the relevant stress or load level and consideration of structural geometry, restraint, pre-existing stress, sensor arrangement, and wave attenuation. The current results should therefore be regarded as a material-level proof of concept rather than a validated structural diagnostic procedure.

### 4.5. Limitations and Further Work

Several limitations should be considered. First, the experimental programme was restricted to one S235 steel and three specimens per thermal condition. The results therefore support an exploratory comparison but are insufficient for establishing universal diagnostic thresholds or robust classification models. A larger dataset is required to quantify between-specimen variability and determine confidence intervals for the proposed descriptors. In addition, detailed chemical-composition data were not available. Because the residual response after high-temperature exposure may depend on the composition and initial metallurgical condition of the steel, the observed trends should be regarded as specific to the investigated S235 product until they are validated for other heats and products.

Second, the AE analysis focused exclusively on counts and cumulative counts. These descriptors are affected by waveform duration, frequency content, acquisition threshold, hit-definition parameters, sensor response, coupling, specimen geometry, and propagation conditions. They should therefore be interpreted as measures of accumulated threshold-crossing activity rather than direct measures of AE energy or the number of physical source events. Further work should determine whether the identified stage-dependent trends are reproduced by hit number, signal energy, amplitude, duration, frequency-related descriptors, and waveform-based classification.

Third, the microstructural characterization presented in this study was qualitative and restricted to representative conditions comprising the as-received material and specimens exposed for 120 min at 700, 900, and 1100 °C. Although these observations demonstrate visible differences in microstructural appearance after thermal exposure, they do not provide a quantitative basis for correlating the stage-resolved AE response with specific microstructural transformations. Future work should therefore combine the present AE methodology with systematic microscopy across all temperature–duration conditions, quantitative grain-size measurements, phase characterization, and hardness measurements.

Fourth, the specimens were exposed under controlled furnace conditions, but their temperatures and cooling rates were not measured directly. The reported temperatures therefore represent nominal furnace conditions. Moreover, real fires involve non-uniform temperature distributions, transient heating and cooling, restraint, thermal stresses, and pre-existing service loads. The unmeasured specimen-specific heating rate represents an additional limitation because heating rate can influence transient temperature gradients and the thermally modified state subsequently obtained after cooling. Accordingly, the present results should be interpreted in relation to the specific furnace exposure procedure used here rather than as independent of the heating history.

Fifth, *N*_cum,75YS_ was evaluated retrospectively using the lower yield load of each specimen. Consequently, it should be interpreted as a mechanically normalized pre-yield descriptor rather than an immediately applicable prospective diagnostic criterion. Future studies should evaluate AE activity at independently defined common stress or strain levels.

The mechanical and AE datasets were synchronized with a temporal resolution of approximately 1 s. A ± 1 s temporal displacement may affect the exact cumulative-count value assigned to a characteristic mechanical point, particularly during periods of rapidly increasing AE activity such as the vicinity of yielding. Because the present analysis relies on stage-resolved cumulative quantities rather than individual event timing, this limitation primarily affects the precise numerical value assigned to the stage boundary. Nevertheless, the magnitude of this effect was not quantified systematically and should be evaluated using higher-resolution hardware synchronization in future studies.

Finally, the indicators were developed using a single specimen geometry, sensor arrangement, threshold, and acquisition system. Their transferability to other geometries and measurement configurations remains to be established. Validation using additional steel grades, larger structural components, alternative AE systems, and realistic post-fire loading scenarios is required before quantitative assessment criteria can be proposed.

## 5. Conclusions

This study examined whether mechanically referenced, stage-resolved AE descriptors provide information on the thermally modified deformation behaviour of S235 steel beyond that obtained from residual strength parameters and final cumulative AE activity. The following conclusions were drawn:

1. Thermal exposure affected not only the final cumulative AE counts but also their distribution over successive stages of tensile deformation. Consequently, the final cumulative count alone did not adequately characterize differences between the investigated thermal histories.
2. Pronounced differences in cumulative AE activity were observed during the mechanically normalized pre-yield stage. After exposure to 900 °C for 120 min, the lower yield strength decreased by approximately 14%, whereas median cumulative counts recorded at 75% of the lower yield load increased by approximately 210% relative to the as-received condition.
3. Relative changes in pre-yield AE counts were not proportional to changes in residual lower yield strength. The AE response therefore did not merely reproduce the magnitude of strength loss but provided complementary information on the evolution of deformation before macroscopic yielding.
4. Similar final cumulative counts could correspond to markedly different AE histories. The 1100 °C/40 min condition exhibited almost the same final cumulative count as the reference condition, despite increased pre-yield activity and a substantially greater contribution of post-UTS activity.
5. The residual AE response varied with the investigated temperature–duration combination rather than with nominal furnace temperature alone, demonstrating that exposure duration should be considered when interpreting the results.
6. The proposed *N*_cum,75YS_ parameter is retrospective because its determination requires knowledge of the specimen-specific lower yield load. It should therefore be treated as a stage-resolved comparative descriptor, not as a directly applicable field threshold.

Overall, mechanically referenced AE analysis provides a useful exploratory framework for comparing thermally modified deformation histories in the investigated S235 steel. Further validation using larger datasets, complementary AE parameters, comparative microstructural characterization, independently defined pre-yield stress levels, additional steel grades, and structural-scale specimens is required before quantitative post-fire assessment criteria can be established.

Future studies should particularly include other commonly used structural steel grades, larger specimen populations, structural-scale elements, higher-resolution synchronization, and prospective AE descriptors referenced to independently defined stress or service-load levels.

## Figures and Tables

**Table 1:** Thermal exposure conditions for S235 structural steel.

Series	Temperature (°C)	Exposure Time (min)	Cooling
S235_0	20	—	Not applicable
S235_700_40	700	40	Air
S235_700_80	700	80	Air
S235_700_120	700	120	Air
S235_900_40	900	40	Air
S235_900_80	900	80	Air
S235_900_120	900	120	Air
S235_1100_40	1100	40	Air
S235_1100_80	1100	80	Air
S235_1100_120	1100	120	Air

**Table 2:** Residual mechanical properties of S235 steel after thermal exposure (mean ± SD, *n* = 3).

Series	σ_YS,L_ [MPa]	σ_YS,H_ [MPa]	σ_UTS_ [MPa]
S235_0	324.14 ± 2.40	351.01 ± 19.43	463.06 ± 1.27
S235_700_40	317.67 ± 4.27	349.19 ± 10.59	447.62 ± 1.76
S235_700_80	293.70 ± 5.33	315.65 ± 6.84	436.41 ± 7.71
S235_700_120	272.50 ± 6.03	319.22 ± 7.01	418.48 ± 6.70
S235_900_40	326.61 ± 2.87	338.88 ± 10.89	458.85 ± 0.80
S235_900_80	302.19 ± 6.56	311.45 ± 8.95	456.48 ± 3.90
S235_900_120	279.81 ± 8.55	294.00 ± 4.49	450.45 ± 6.73
S235_1100_40	266.18 ± 4.20	273.15 ± 2.79	425.09 ± 3.79
S235_1100_80	232.32 ± 5.84	-	390.14 ± 2.40
S235_1100_120	231.01 ± 11.51	240.53 ± 8.95	382.04 ± 10.14

**Table 3:** Median cumulative AE counts recorded by channel 2 at characteristic stages of tensile deformation (*n* = 3).

Series	*N*_cum,75YS_	*N*_cum,YS_	*N*_cum,UTS_	*N*_cum,f_
S235_0	57,552	63,142	289,381	292,460
S235_700_40	75,798	79,948	219,014	253,840
S235_700_80	47,184	48,285	233,283	241,707
S235_700_120	71,469	72,849	162,872	185,165
S235_900_40	76,148	85,750	326,045	370,720
S235_900_80	68,825	101,298	297,268	326,462
S235_900_120	178,636	203,479	357,790	377,598
S235_1100_40	95,022	102,911	172,156	292,180
S235_1100_80	87,346	93,655	232,508	238,851
S235_1100_120	81,776	86,917	258,602	331,415

**Table 4:** Individual and median cumulative AE counts recorded at 75% of the lower yield load.

Series	Specimen 1	Specimen 2	Specimen 3	Median	Min–Max	Range Relative to Median [%]
S235_0	57,552	64,126	50,873	57,552	50,873–64,126	23.0
S235_700_40	69,231	87,264	75,798	75,798	69,231–87,264	23.8
S235_700_80	55,791	43,217	47,184	47,184	43,217–55,791	26.6
S235_700_120	71,469	64,992	76,934	71,469	64,992–76,934	16.7
S235_900_40	89,357	70,114	76,148	76,148	70,114–89,357	25.3
S235_900_80	57,462	76,984	68,825	68,825	57,462–76,984	28.4
S235_900_120	178,636	201,583	166,417	178,636	166,417–201,583	19.7
S235_1100_40	110,814	81,739	95,022	95,022	81,739–110,814	30.6
S235_1100_80	79,208	103,671	87,346	87,346	79,208–103,671	28.0
S235_1100_120	89,731	68,695	81,776	81,776	68,695–89,731	25.7

## Data Availability

The original contributions presented in this study are included in the article. Further inquiries can be directed to the corresponding author.
